# Sex-related differences in cardiovascular inflammation and metabolomics in a humanized transgenic mouse model of celiac disease

**DOI:** 10.1038/s41598-026-45481-6

**Published:** 2026-03-26

**Authors:** Aline Pesi, Simon Lange, Fabian Schmitt, Manjusha Neerukonda, Michelle Wiegel, Theodora Petridou, Henning Ubbens, Lea Strohm, Dominika Mihalikova, Ivana Kuntic, Marin Kuntic, Philipp Lurz, David Leistner, Karin Keppeler, Elena F. Verdu, Thierry Schmidlin, Andreas Daiber, Detlef Schuppan, Sebastian Steven

**Affiliations:** 1 Institute of Translational Immunology and Research Center for Immunotherapy, University Medical Center of the Johannes Gutenberg University, Mainz, Germany; 2 Department of Cardiology, University Medical Center of the Johannes Gutenberg University, Mainz, Germany; 3 Institute of Immunology, University Medical Center of the Johannes Gutenberg University, Mainz, Germany; 4 Farncombe Family Digestive Health Research Disease Institute, Department of Medicine, McMaster University, Hamilton, Canada; 5 Research Center for Immunotherapy (FZI), University Medical Center of the Johannes-Gutenberg University, Mainz, Germany; 6 Division of Gastroenterology, Beth Israel Deaconess Medical Center, Harvard Medical School, Boston, MA USA; 7 Department for Cardiology, Goethe University, Frankfurt, Germany; 8 German Center for Cardiovascular Research (DZHK), Partner Site Rhine-Main, Mainz, Germany

**Keywords:** Diseases, Gastroenterology, Immunology, Medical research, Physiology

## Abstract

Celiac disease (CeD) is an immune-mediated disorder driven by dietary gluten, characterized by intestinal, such as diarrhoea and malabsorption, and many extraintestinal manifestations. Epidemiological studies have linked untreated CeD to an elevated risk of cardiovascular complications. In this study, CeD induced in humanized transgenic male NOD.DQ8 mice resulted in vascular dysfunction, cardiovascular oxidative stress, and systemic as well as vascular inflammation. In contrast, female NOD-DQ8 mice developed comparable CeD-specific small intestinal pathology upon gluten exposure but were protected from vascular dysfunction and cardiovascular inflammation. Compared with females, male CeD mice displayed pronounced dysregulation of cholesterol metabolism, activation of the kynurenine pathway, and mast cell activation in perivascular tissues. Pharmacological reduction of estrogen with the aromatase inhibitor letrozole partly impaired vascular dilatation in female CeD mice. These findings underscore the importance of considering sex-specific manifestations of CeD with its associated systemic inflammation in preclinical and clinical research.

**Supplementary Information:**

The online version contains supplementary material available at 10.1038/s41598-026-45481-6.

## Introduction

Celiac disease (CeD) is a small intestinal autoimmune-like disease driven by ingestion of gluten which is the main protein component of wheat and related cereals^1^. The intestinal histological hallmark of CeD is villous atrophy and intraepithelial lymphocytosis. Individuals with untreated CeD can present with a wide spectrum of symptoms, ranging from classical gastrointestinal manifestations including diarrhoea, bloating, abdominal pain, and malabsorption to a range of extra-intestinal symptoms and autoimmune diseases, including type 1 diabetes and autoimmune thyroiditis^1–5^. The human leukocyte antigen (HLA) haplotypes DQ2 and DQ8 confer the genetic predisposition to develop CeD but are not sufficient on their own. Although 30–40% of individuals in most populations carry HLA-DQ2 or HLA-DQ8, only ~ 1% worldwide develop CeD. These HLA molecules selectively present gluten-derived peptides to intestinal antigen-presenting cells, particularly after deamidation by the ubiquitous enzyme tissue transglutaminase in the lamina propria, thereby driving T cell activation and intestinal inflammation^1–5^. The only approved therapy for CeD is life-long adherence to a strict gluten-free diet (GFD), but mechanism-based pharmacological therapies are evolving^6,7^. CeD shows a higher prevalence in women (female-to-male ratio ~ 2:1), with increased risk of infertility and adverse pregnancy outcomes^8,9^. Furthermore, women with CeD frequently experience delayed menarche and an earlier onset of menopause^10^.

Regarding cardiovascular implications, Ludvigson et al*.* observed an increased risk for ischaemic heart disease in untreated male and female CeD patients^11^. In a previous publication of our group the cardiovascular effects of active CeD in the humanised HLA-DQ8 transgenic NOD-DQ8 CeD mouse model were assessed, demonstrating that active CeD in these mice lead to increased oxidative stress, vascular inflammation and endothelial dysfunction^12^. However, these studies relied exclusively on male mice, reflecting a long-standing practice in biomedical research. For many years, female mice were largely excluded under the assumption that their estrous cycle would increase data variability, thereby introducing a systematic sex bias.^13–16^.

We therefore employed a model of celiac disease (CeD) to investigate gender-specific differences. Male and female mice with active gluten-induced enteropathy were examined with respect to vascular dysfunction, oxidative stress, and inflammation. To gain deeper mechanistic insights, we additionally assessed the effects of estrogen inhibition in female mice and analyzed intestinal and vascular cytokine profiles, as well as lipid and cholesterol signaling and metabolism. Our findings highlight the importance of including both sexes in preclinical research and of systematically evaluating extra-intestinal manifestations of CeD in clinical practice.

## Materials and methods

### Animal housing

All studies have been approved by the appropriate ethics committee. The performed breeding, housing and study of NOD-DQ8 mice was approved by the local authorities, namely Landesuntersuchungsamt Rhineland Palatinate, Koblenz, Germany (permit number: 23 177-07/G 20-1-61). All experiments were conducted in accordance with the established guidelines and regulations. This study is reported in accordance with the ARRIVE guidelines (https://arriveguidelines.org). Mice of both sexes were included into the experimental phase when aged 8–12 weeks with a minimum weight of 20 g. With a 12 h dark/light cycle, animals were housed in ventilated animal cabinets as groups of either male- or female-only littermates. All mice had ad libitum access to fresh drinking water and gluten-free chow until chow was changed during the experimental phase. The mouse line used in this study was obtained from Prof. Elena Verdu (Mc Master University) and maintained in our facility.

### Feeding experiments and organ harvest

Male and female mice were randomly separated in two groups, gliadin and control (Ctr). Diet of the gliadin group was changed to a gluten-containing diet (GCD) for two weeks while the control group was fed with the gluten-free diet (GFD) further on. Additionally, both groups were sensitised with pepsin-trypsin digested and deamidated zein (Ctr) or gliadin (gluten) in combination with cholera toxin via oral gavage on day 0, 7 and 14. Composition of the chows as well as the preparation of zein and gliadin used for gavage was performed as described in ^12^. Animals were killed on day 15 after diet change by applying an overdose of anaesthetics (i.p. injection of 120 mg/kg BW ketamine and 16 mg/kg BW xylazine). Blood sampling was performed by cardiac puncture before organs of the abdomen and thorax were harvested. Serum levels for LDL-, HDL- and total cholesterol were analysed from these blood samples in the Institute of Clinical Chemistry and Laboratory Medicine of the University Medical Center Mainz, Germany, following standard protocols for enzymatic colorimetric testing of serum cholesterol. Aorta and adjacent tissues were sampled as one and dissected manually under use of binocular loupes. Depending on the further use of the tissues, specimens were either snap-frozen in liquid nitrogen, fixed in 4% paraformaldehyde or used fresh for same-day analysis.

### Isometric relaxation analysis

Endothelial-dependent and -independent relaxation capability analysis of aortic rings was executed in an organ bath set-up^12^. Aortic rings of 4 mm from the thoracic section were cleaned from fat and adhesive tissue before applying approx. 80% of the maximum constriction by exposition to potassium chloride in the organ bath. Via force gauges, the occurring relaxation of the pre-constricted tissue under rising concentrations of the relaxation-inducing compounds acetylcholine (endothelium-dependent) and nitroglycerine (endothelium-independent) was monitored.

### Real-time quantitative polymerase chain reaction (RT-qPCR) – TAQMAN

Snap-frozen tissue samples were homogenized using a Tissue Lyser II (Qiagen, Germany). Isolation of mRNA was achieved by the acid guanidium thiocyanate-phenol–chloroform extraction method ^17^. Extracted mRNA was analysed by RT-qPCR in a Quantstudio 3 PCR machine under use of TaqMan primer and the TaqMan Fast Virus 1-Step MasterMix (all Thermo Fisher Scientific, MA, USA). The applied TaqMan primer are *Vcam1* (Mm00449197_m1), *Tnfα* (Mm00443259_g1), *iNOS*/Nos2 (Mm00440485_m1), and *Tbp* (Mm00446973_m1). Threshold cycle values were normalised to TATA-box binding protein (*Tbp*) expression and final expression levels were calculated by the ΔΔCt method.

### ***Real-time quantitative polymerase chain reaction (RT-qPCR) – SYBR green***

RNA from duodenum was extracted using the Monarch RNA cleanup kit (#T2050; NEB, Ipswich, MA, US). Quantitative polymerase chain reaction (qPCR) was carried out using exon–exon boundary-spanning primer sequences and the SYBR Green methodology using the Luna® Universal One-Step RT-qPCR Kit (E3005E, NEB, Ipswich, MA, US) on a Step One Plus sequence amplification system (Applied Biosystems, Foster City, CA, USA). The relative mRNA expression of the tested gene was normalized to beta actin expression. Results were calculated by the ΔΔCt method.

### Intestinal histology

Distal jejunal fixed samples were cut to 5 µm sections and were subsequently stained with anti-CD8 (ab209775, abcam, Cambridge, Cambridgeshire, UK) and anti-B220 (CD45R) antibodies (MA1-70098, invitrogen, Carlsbad, California, USA) and counterstained with hematoxylin. 20 × images of stained sections were taken using the Leica XM5 microscope and stitched together to get a whole-section image. The amount of lymphocytes was counted using the Image J Fiji counter and normalized to the nuclei count per analyzed villus.

### LC–MS analysis

Metabolite extracts derived from plasma of male and female NOD-DQ8 mice were prepared by adding 280 µl of –20 °C cold lysis buffer (80% methanol / 20% water) to 25 µl of plasma for protein-precipitation followed by centrifugation at 24.400 g for 30 min. 150 µl of supernatant was transferred to a new tube and evaporated under constant nitrogen flow. Dried metabolites were resuspended in 100 µl LC–MS grade water. 1 µl of the metabolite extracts were subjected to analysis using liquid chromatography-mass spectrometry (LC–MS) using an Agilent 1290 Infinity II coupled to a SCIEX ZenoTOF 7600. Metabolites were separated on a Phenomenex Kinetex F5 2.6 µm (2.1 mm × 150 mm) column using a linear gradient from 0.1% formic acid (FA) in water to 0.1% FA in 95% acetonitrile (ACN) / 5% water. MS spectra were acquired in positive ion mode using a precursor ion mass range of 70 – 1000 m/z. Per cycle top 11 precursor ions were subjected to fragmentation using data-dependent acquisition. MS/MS spectra were recorded in the mass range of 40–1000 m/z, with Zeno pulsing enabled and collision induced dissociation performed at a collision energy of 35 V ± 15 V. LC–MS data files were then processed in Skyline (version 22.2.0.527). XICs of [M + H] + , [M + K] + and [M + Na] + adducts were generated using the orthogonal information of precursor m/z mass accuracy and explicit RT information included in the transition list, containing our in-house recorded spectral library of authentic reference standards as described in Schmitt et al.^18^. Area under the curve values were exported from Skyline as CSV and utilized in subsequent statistical analysis.

### Statistics

The statistic analysis was conducted by GraphPad Prism v10.1.0 (GraphPad Software Inc., MA, USA). The testing for outliers was performed using the ROUT Test with Q = 1 as level of significance. After the exclusion of outliers, normal distribution of the data was tested by Shapiro–Wilk test. A One-way ANOVA was applied for all RT-qPCR results, histology analysis, and body weight gain. Isometric tension studies were analysed by a Two-way ANOVA. Metabolome analysis was carried out using the MetaboAnalyst 6.0 platform^19^ using one factor Statistical Analysis for peak intensities. Normalization was done by volume. Bonferroni’s correction was applied to correct for Type I errors and all tests were assumed to be two-tailed.

## Results

### Intestinal inflammation in male and female NOD.DQ8 mice after gluten exposure

**Fig. 1:**
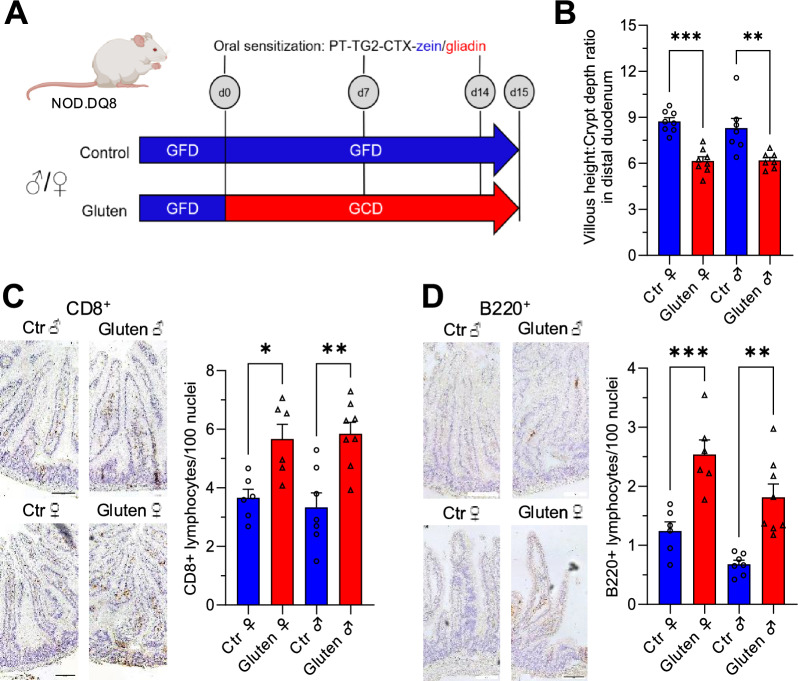
Exposure to gluten induces active celiac disease (CeD) in both male and female NOD.DQ8 mice. (**A**) Male and female NOD.DQ8 littermates were housed in sex-separated cages and maintained on the zein containing gluten-free diet (GFD). At 8–12 weeks of age, mice were divided into a gluten containing diet (GCD) and GFD control group, accompanied by oral sensitization to their respective antigens. Oral sensitization was performed via gavage with gliadin (PT-TG2-CTX-gliadin) or with pepsin-trypsin-digested zein (PT-TG2-CTX-zein). Gavage was administered on day 0, 7 and 14. Tissues and blood were collected on day 15. (**B**) Both sexes developed active CeD on GCD, indicated by a reduced villous height-to-crypt depth ratio. (**C**,**D**) CD8⁺ and CD45R⁺/B220⁺ lymphocyte counts in duodenal histological sections were significantly increased in both sexes on the GCD (highlighted in brown in representative images). (**A**) created with BioRender. (**B**–**D**) Bar graphs show the arithmetic mean and SD. One-way ANOVA, n = 6–8. Scale bar in representative images = 100 µm. *p < 0.05; **p < 0.01; ***p < 0.001.

Male and female NOD.DQ8 mice were kept on a gluten-free diet (GFD) from weaning. At 8–12 weeks of age, mice were randomized to two groups. The control group received gluten-free chow containing the corn protein zein. The gluten group received chow in which the zein fraction was replaced by gluten, while all other components remained identical. Both diets were given for two weeks. In addition, mice were orally gavaged on days 0, 7, and 14 with either zein or deamidated gliadin, both pretreated by pepsin/trypsin digestion. Gliadin represents the major immunogenic protein fraction of gluten (Fig. 1A).

Mice of both sexes on the gluten containing diet (GCD) developed intestinal inflammation characteristic of human CeD, such as a decreased villus height to crypt depth ratio (VH:CrD) (Fig. 1B) and increased intraepithelial lymphocytosis, including significantly elevated numbers of intraepithelial CD8^+^ T cells and of lamina propria B220^+^ B cells (Fig. 1C,D).

### Cardiovascular effects of active CeD in male and female NOD.DQ8 mice

**Fig. 2:**
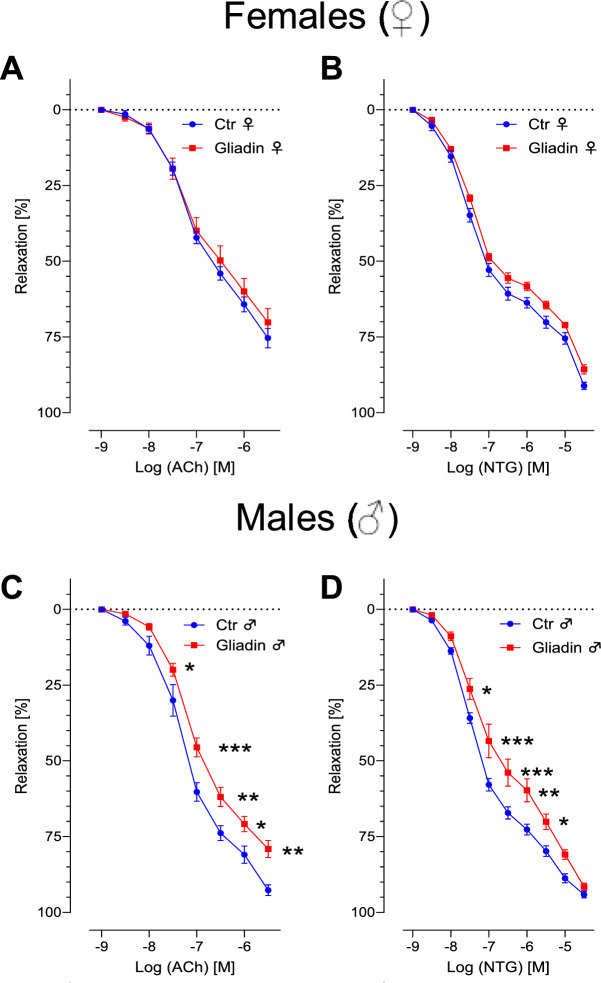
Vasodilation of NOD.DQ8 is impaired in male but not in female mice with active CeD. (**A**,**C**) Endothelium-dependent relaxation capability of aortic rings was tested in organ baths using rising concentrations of acetyl-choline (ACh). (**B**,**D**) Nitroglycerin (NTG) was applied to test the endothelium-independent relaxation capability using the same aortic rings. (**A**–**D**) Data shown as arithmetic mean and SD. Two-way ANOVA, n = 11–16; * is p < 0.05; ** is p < 0.01; ***p < 0.001.

Vascular function was assessed by isometric tension studies on aortic rings in an organ bath. Endothelium-dependent relaxation was tested with rising concentrations of acetylcholine (ACh). Endothelium-independent relaxation was examined using nitroglycerine (NTG). Male NOD.DQ8 mice with active CeD induced by GCD developed severe vascular dysfunction, with impaired relaxation in response to both ACh and NTG (Fig. 2A,B). This dysfunction was marked by a significant reduction in endothelial relaxation and a diminished NTG response. In contrast, female NOD.DQ8 mice with active CeD showed normal vascular reactivity, with preserved relaxation to both ACh and NTG. (Fig. 2C,D).

**Fig. 3:**
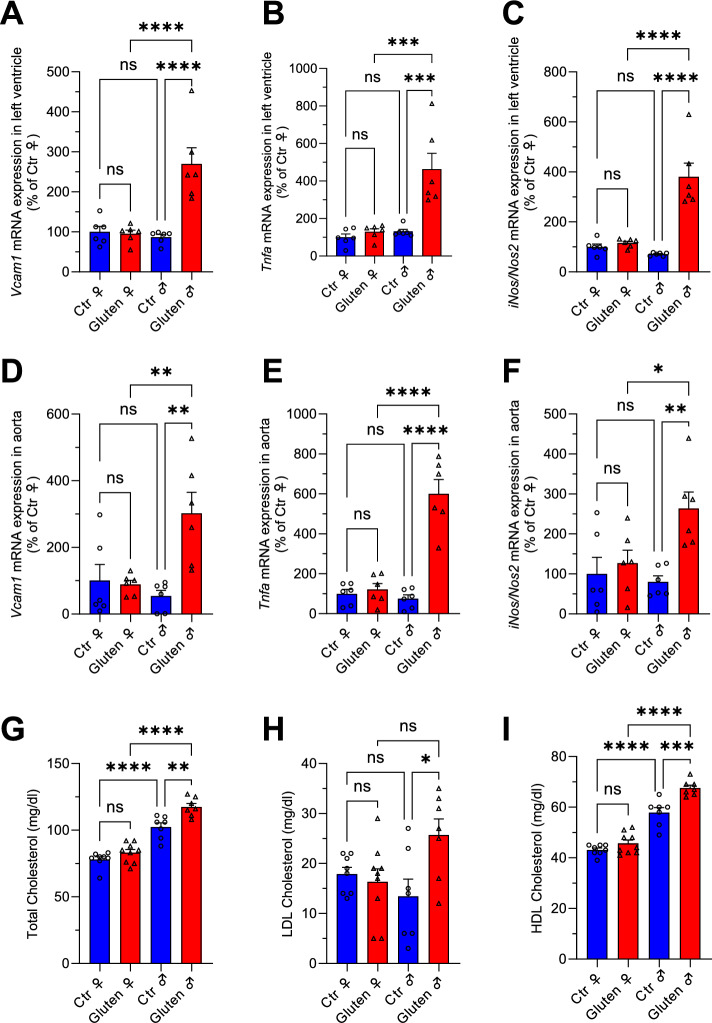
Cardiovascular tissues and cholesterol levels of female NOD-DQ8 mice show no inflammatory response to active CeD. (**A**–**C**) Transcript levels of biomarkers for inflammation and oxidative stress were analysed in tissue of the left ventricle by qPCR. Vascular cell adhesion molecule 1 (VCAM1), tumor necrosis factor alpha (TNFα), and inducible NO synthase (iNos/Nos2) were significantly elevated on mRNA level in males. Heart tissue of NOD-DQ8 females showed no differences in the tested groups. (**D**–**F**) The same biomarkers were tested in aortic tissue, too. Glutenexposed males showed a severe increase of inflammatory markers. Again, females did not develop such a phenotype. (**G**–**I**) By colorimetric enzymatic testing, the same pattern was observed regarding levels of serum cholesterol. Gluten-fed males displayed increased cholesterol levels while females showed no diet-induced alterations. (**A**–**I**) Bar graphs show the arithmetic mean and SD. One-way ANOVA, n = 6–9; * is p < 0.05; ** is p < 0.01; *** p < 0.001; **** is p < 0.0001.

To investigate cellular and immunological mechanisms underlying the observed sex differences, we quantified the expression of inflammatory markers by qPCR in left ventricular and aortic tissue. Transcriptional levels of vascular cell adhesion molecule-1 (VCAM1), tumor necrosis factor-α (TNFα), and inducible nitric oxide synthase (iNOS/Nos2) were significantly elevated in male mice, but not in females (Fig. 3A–F). These findings align with the vascular dysfunction observed exclusively in males and support the absence of vascular alterations in females under comparable conditions of gluten-induced intestinal inflammation. To further explore potential contributors to male-specific cardiovascular impairment, we analysed serum lipid profiles. Male mice displayed significant increases in total and LDL cholesterol, whereas females exhibited no such changes. Instead, female mice showed a mild, non-significant increase in HDL cholesterol (Fig. 3G–I).

Inhibition of estrogen synthesis in female NOD.DQ8 mice by administering the aromatase inhibitor letrozole (1 mg/kg BW by i.p. injection) resulted in an endothelium-independent vascular dysfunction, as evidenced by isometric tension studies, while endothelium-based relaxation remained unaffected. Notably, the body weight was not altered in the tested groups (Supp. Fig. 1D−F).

### Male but not female NOD.DQ8 mice show increased inflammation and activation of cholesterol-related pathway in pVAT

**Fig. 4:**
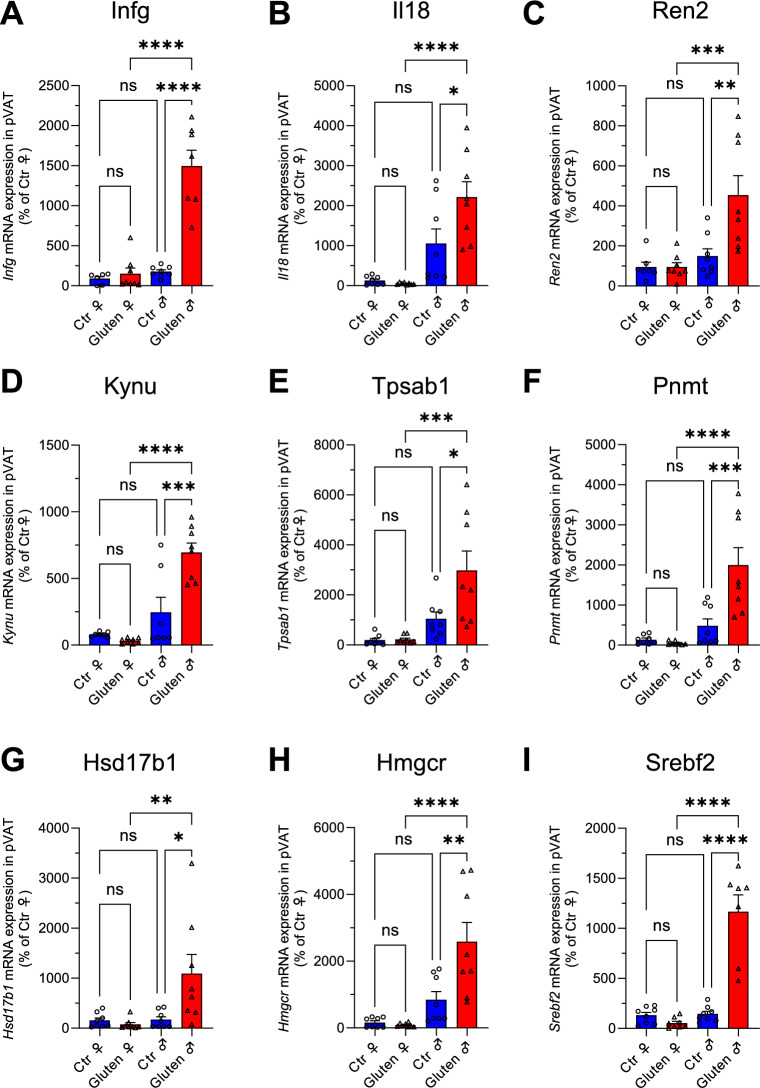
Perivascular adipose tissue (pVAT) of male gluten-fed NOD.DQ8 mice express high levels of inflammatory markers and indicate activated cholesterolrelated pathways. (**A**–**C**) Investigated pVAT of gluten-exposed male NOD. DQ8 mice displayed increased inflammation-associated transcript levels of interferon gamma (Ifnγ), interleukin 18 (Il18) and renin 2 (Ren2). (**D**–**F**) The mRNA levels of the stressrelated marker phenylethanolamine N-methyltransferase (Pnmt), kynureninase (Kynu) and the mast cell-associated tryptase alpha/beta-1 (Tpsab1) were found elevated in pVAT of males only, too. (**G**–**I**) Furthermore, the transcript expression of the cholesterol pathway regulators 17β-hydroxysteroid dehydrogenase 1 (Hsd17b1), 3-hydroxy-3methyl-glutaryl-coenzyme A reductase (Hmgcr) and sterol regulatory element-binding protein 2 (Srebf2) were measured elevated just in males fed a gluten diet. Female mice did not develop any significant alteration regarding the shown targets. (**A**–**I**) Bar graphs show the arithmetic mean and SD. One-way ANOVA, n = 7–8; * is p < 0.05; ** is p < 0.01; *** p < 0.001; **** is p < 0.0001.

Gluten exposure in NOD.DQ8 mice led to pronounced, sex-specific changes in *perivascular adipose tissue* (*pVAT*). Only male mice displayed increased transcript levels of *interferon-γ* (*Ifng*), *interleukin-18* (*Il18*), and *renin-2* (*Ren2*) (Fig. 4A–C). Similarly, transcripts of mast cell– and stress-associated enzymes, including *kynureninase* (*Kynu*), *tryptase* (*Tpsab1*), and *phenylethanolamine N-methyltransferase* (*Pnmt*), were elevated exclusively in males (Fig. 4D–F). The same male-specific pattern was observed for sterol regulatory *element-binding protein-2* (*Srebf2*), *HMG-CoA reductase* (*Hmgcr*), and *17β-hydroxysteroid dehydrogenase-1* (*Hsd17b1*), key regulators of cholesterol metabolism and steroid synthesis (Fig. 4G–I). In contrast, female mice showed no significant changes in these parameters following gluten exposure.

### Sex-dependent differences in CeD associated alterations of metabolic profiles in plasma

**Fig. 5:**
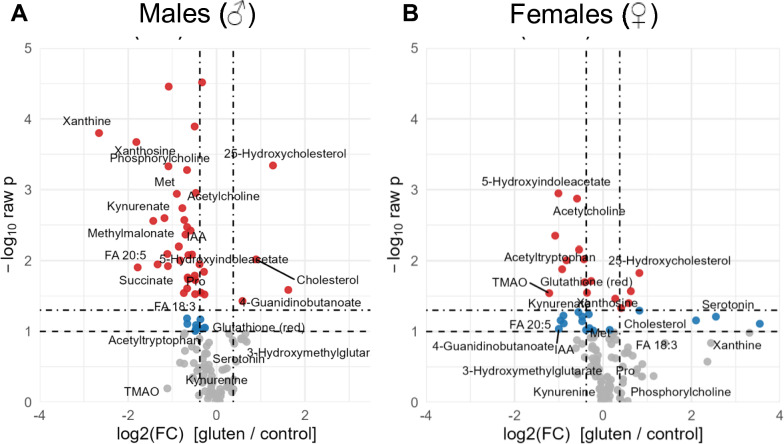
Male NOD.DQ8 mice undergo increased metabolic shift when challenged with active CeD. (**A**,**B**) Mass spectrometry-based investigation on the metabolic profiles of plasma revealed differences between male and female NOD.DQ8 mice. Male and female gluten-treated mice showed alterations in their metabolic profile when compared to their control littermates of the same sex as visualised in the volcano plot. In the male mice, 30 of the tested metabolites were altered significantly as just 13 metabolites appeared significantly affected by the gluten exposition in females. The level of significance (p < 0.05) is illustrated by the thicker lines. Significance level and fold change are illustrated by the size and colour of the dot representing each metabolite. For better visibility, only significantly altered metabolites are shown by name. n = 6–8 per group. Metabolome analysis was carried out using the MetaboAnalyst 6.0 platform^45^ using one factor Statistical Analysis for peak intensities. Normalization was done by volume. Bonferroni’s correction was applied to correct for Type I errors and all tests were assumed to be two-tailed. Significance levels were defined and illustrated as follows: p > 0.05 is not significant (ns), p < 0.05 is *, p < 0.01 is **, p < 0.001 is ***, p < 0.0001 is ****.

Given the observed upregulation of cholesterol pathway-associated genes in male NOD.DQ8 mice upon gluten feeding, we next performed an untargeted metabolic profiling of plasma samples using LC–MS based metabolomics (Fig. 5). Relative metabolite abundances were compared between active CeD and control mice of both sexes, and significant differences were defined as fold change (FC) > 1.5. Results were visualized as volcano plots to highlight both the magnitude and significance of metabolic changes (Fig. 5).

Male mice exhibited a markedly broader metabolic response to gluten exposure than females. Specifically, 30 of 141 detected metabolites were significantly altered in males with active CeD compared to their non-celiac controls, whereas only 13 metabolites reached significance in females. This sex-specific difference underscores a more profound metabolic disruption in males.

Upon gluten exposure, plasma serotonin increased more in female than in male mice, whereas plasma kynurenate (a metabolite with reported neuroprotective properties) was significantly lower in male CeD mice than in male controls. Additionally, both sexes demonstrated increased levels of 25-hydroxycholesterol, consistent with a disturbance of cholesterol biosynthesis (Supp. Fig. 4). However, the magnitude of change was more pronounced in males, who also showed elevated mevalonate, a central precursor metabolite in the cholesterol synthesis pathway. These findings indicate that gluten-induced CeD triggers a male-specific amplification of metabolic disturbances, particularly within the cholesterol biosynthetic cascade.

## Discussion

Celiac disease (CeD) is a gluten-driven immune-mediated enteropathy with clinically relevant extraintestinal manifestations, yet the mechanisms linking intestinal inflammation to cardiovascular pathology remain incompletely defined. This study investigated sex-specific differences in a human transgenic rodent model of celiac disease (CeD), a well-characterized small intestinal inflammatory disorder triggered by dietary gluten. The NOD.DQ8 mouse model provides a platform to examine both intestinal and extraintestinal manifestations of CeD, including cardiovascular complications^12^. We demonstrate a pronounced sexual dimorphism: despite comparable gluten-induced small intestinal pathology in males and females, only males developed impaired endothelium-dependent and -independent vasorelaxation accompanied by vascular and myocardial inflammatory activation, a pro-inflammatory transcriptional signature in perivascular adipose tissue (pVAT), and a broader disturbance of the plasma metabolome. Female mice were largely protected, and pharmacological inhibition of estrogen synthesis partially attenuated and thus worsened vascular relaxation, implicating sex hormones as modulators of the gut–vascular axis. Both male and female mice showed comparable gluten-induced pathology, with reduced villus height-to-crypt depth ratios and increased CD8⁺ cytotoxic T cell and B220⁺ B cell infiltration (Fig. 1). In contrast male but not female mice exhibited severe vascular dysfunction and elevated inflammatory markers across aortic tissue, perivascular adipose tissue (pVAT), and myocardium (Figs. 2, 3, Supplementary Fig. 2), consistent with previous findings^12^. Only a modest reduction in endothelial relaxation compared to control males was observed in females (Fig. 2A,C), in line with reports from other inflammatory models like hindlimb ischemia and colitis, implicating significantly impaired vasodilation and reactive oxygen species (ROS) generation only in males^20,21^.

Our data may suggest that male but not female mice experience widespread pro-inflammatory cell activation and infiltration spreading from the intestinal system into the pVAT (Fig. 4). The elevated transcript levels of *IL18* observed only in male pVAT samples (Fig. 4B) reflect a substantial component of the inflammasome pathway^23^, which triggers caspase-1, resulting in pyroptosis, a lytic form of cell death that further intensifies the pro-inflammatory immune response^24^. Notably, pVAT samples of gluten-exposed males also exhibited signs of non-classical mast cell activation, characterized by the upregulated *Trpa/Trypb* and *Mrgprx2* genes (Fig. 4, Supplementary Fig. 3), whereas these transcripts were not elevated in female pVAT. Mast cells are known to release histamine, proteases, leukotrienes and cytokines promoting leukocyte rolling, adherence and emigration through the vessel wall into the adjacent tissue^22^. Via these recruiting features and the resulting immigration of leucocytes into the vessel wall, activation of mast cells could be another cofactor for the reduced dilation capability observed in males^23–25^. We further identified upregulated expression of transcripts encoding the CeD-typical cytokine IFNγ in male but not in female pVAT tissue of our mice with CeD. Since the intestinal immune response of CeD is highly dominated by IFNγ, this may suggest the presence of potentially gut-derived gluten-induced T cells attracted to this location^7^. It also suggests an active role of (perivascular) adipose tissue in disseminating and attracting (intestinal) inflammation. The close proximity of pronounced inflammation in pVAT is a plausible contributing factor for the impaired relaxation capability in the aorta in males^26^.

Another important factor in cardiovascular diseases is the presence of excessive cholesterol levels in plasma, with elevated LDL-cholesterol considered as particularly dangerous^27–29^. In our mouse model of CeD, male mice exhibited increased circulating total and LDL cholesterol alongside upregulation of *Srebf2* and *Hmgcr* in pVAT, consistent with activation of cholesterol biosynthetic programs in inflamed perivascular tissue (Fig. 3G/H/I, Fig. 4I). SREBF2 is a master transcription factor being activated during inflammation and leading to the enhanced production of cholesterol. There is a link between NLRP3 inflammasome activation and maturation of SREBF2 in macrophages^30^. Transcripts of the HMG-CoA reductase, the rate-limiting enzyme in cholesterol biosynthesis and the mevalonate pathway, were also significantly increased only in male CeD mice, further supporting an upregulated cholesterol production in adipocytes and immune cells (Fig. 4H). Our data support a role of excessive (LDL) cholesterol synthesis along with the pro-inflammatory vascular immune response in male CeD mice. These findings are notable because lipid alterations in human CeD have been reported as heterogeneous, potentially reflecting differences in disease stage, degree of malabsorption, diet, and inflammation. Recent meta-analyses support a modest but significant increase in cardiovascular disease risk in CeD overall, despite substantial heterogeneity across cohorts^31^. In another study, triglycerides and HDL increased under the gluten-free diet, but may be linked to improved nutrient malabsorption^32^. However, only few studies investigated effects of CeD on cholesterol levels in humans with active CeD on a gluten containing diet. Salardi et al. demonstrated elevated LDL levels in untreated CeD children with concomitant Type 1 Diabetes mellitus^33^. CeD-associated chronic intestinal inflammation and barrier dysfunction can promote systemic inflammatory signaling, which is known to impair hepatic LDL receptor-mediated clearance and increase circulating LDL levels. Our mouse data support the concept that inflammation-driven mechanisms alone are sufficient to elevate LDL in CeD^34,35^. Moreover, pharmacological inhibition of cholesterol synthesis with a HMG-CoA reductase inhibitor suppressed dendritic cell activation^36^. These findings are well in line with the molecular and mechanistic data in our CeD model, warranting further clinical research to investigate cholesterol levels in male and female patients with CeD and other autoimmune diseases. In conjunction with the heterogeneous epidemiologic evidence regarding cardiovascular risk in celiac disease, we propose that disease stage may reconcile discrepant lipid findings: an early, inflammation-dominant phase like in this acute setting, may strongly engage the NLRP3-SCAP/SREBP2-HMGCR axis and elevate circulating LDL, whereas long-standing untreated disease with villous atrophy and malabsorption is associated with hypocholesterolemia that could mask or counterbalance lipid-driven cardiovascular risk signals. Prior studies on this mouse model observed no difference in zein- and gluten-fed males concerning the non-fasting blood glucose levels^12^. This parameter was not investigated in the female littermates but could be a suitable candidate for future studies since elevated non-fasting glucose levels are associated with an increased hazard rate for myocardial infarction and ischemic heart disease^37–39^.

Inflammation-induced imbalances in the kynurenine pathway have been associated with coronary atherosclerotic disease. Downstream products of the kynurenine pathway, such as quinolinic acid and subsequent ROS generation, have been linked to coronary artery calcification in clinical trials^40^, while kynurenic acid, a neuroprotective compound from the alternative kynurenine degradation pathway, has protective effects^41^. However, kynurenic acid can undergo a pathway involving kynurenin-3-monooxygenase (KMO) and kynureninase (KYNU) to produce neurotoxic quinolinic acid, subsequently initiating the release of ROS which are usually detrimental if released at cardiovascular sites^42,43^. We showed elevated transcripts for KYNU only in pVAT of male with enterophathy CeD mice compared with controls (Fig. 4D). While we were not able to detect quinolinic acid in our metabolomics experiments, we detected a significant downregulation of the protective metabolite kynurenic acid in CeD plasma samples of both sexes, but more pronounced in plasma samples of gluten exposed males (Fig. 5)^44^. This pathway and related metabolites might be another part of the puzzle to understand inflammation-mediated cardiovascular impairment during CeD.

We investigated further molecular pathways to explain the observed gender difference in CeD related vascular inflammation and dysfunction and examined several sex-hormone-related enzymes. We identified elevated transcript levels for 17β-hydroxysteroid dehydrogenase 1 (17β-HSD1) exclusively in pVAT of male CeD mice (Fig. 4G). 17β-HSD1 has a double function: it catalyses the conversion of estron and estradiol as of 4-androstenedione to testosterone, acting as an important regulator of steroid hormone production, being also required for male fertility^45,46^. Prenatal testosterone exposure was shown to correlate with hypertension in females, and to a greater extend in male rodent offspring paralleled by upregulation of transcripts encoding 17β-HSD1 in testes^47^. Possibly, in this model, the regulatory inflammatory axis is coupled with the steroid hormone synthesis pathway.

In the present study, we detected substantial cardiovascular alterations in response to gluten exposition across both sexes. Notably, only male mice exhibited deteriorations of the cardiovascular health when active CeD was triggered. However, we were not yet able to clearly identify the responsible metabolite(s) or pathway(s) that is/are mainly responsible for these altered phenotypes. Regarding cardiovascular health, a mouse model for pancreatic and liver cancer demonstrated differences in the heart rate variability between both sexes^48^. Furthermore, a translational approach comparing human and murine heart tissue revealed the important role of estrogen in the context of mitochondrial function and thereby overall cardiovascular health^49^. Consequently, estrogen and its precursor molecules play crucial roles as regulators of energy metabolism^43–45^ and inflammation^46,47^. In letrozole-treated female CeD mice, we observed an impairment of endothelium-independent relaxation (Supplementary Fig. 1). Future studies on the NOD.DQ8 mouse model should comprehensively investigate estrogen- or testosterone-mediated mechanisms and the microbiome’s role in both sexes.

In summary, our study identifies sex as a major determinant of cardiovascular involvement in experimental CeD in our humanized mose model. Male mice exhibited a coordinated phenotype of perivascular inflammation, immunometabolic activation of cholesterol pathways, and broader systemic metabolic disruption that coincided with vascular dysfunction, whereas females were largely protected in an at least partly estrogen-dependent manner. Our data suggest that male-specific alterations in cholesterol/steroid metabolism, inflammasome activation, and kynurenine pathway imbalance drive this susceptibility. These results provide a mechanistic framework for sex- and stage-dependent heterogeneity in CeD-associated cardiovascular risk and support more systematic consideration of cardiovascular assessment and risk factor profiling in CeD, stratified by sex and hormonal status, and may serve as basis to study the disease modifying role of these factors in human subjects with CeD.

## Supplementary Information

Supplementary Information.

## Data Availability

The datasets generated during and analysed during the current study are available from the corresponding author on reasonable request.
