# Supplementary material for: Sex-related differences in cardiovascular inflammation and metabolomics in a humanized transgenic mouse model of celiac disease
Source: Sci Rep. 2026 Mar 26;16:10509. doi: 10.1038/s41598-026-45481-6 (PMC13031320; doi:10.1038/s41598-026-45481-6)

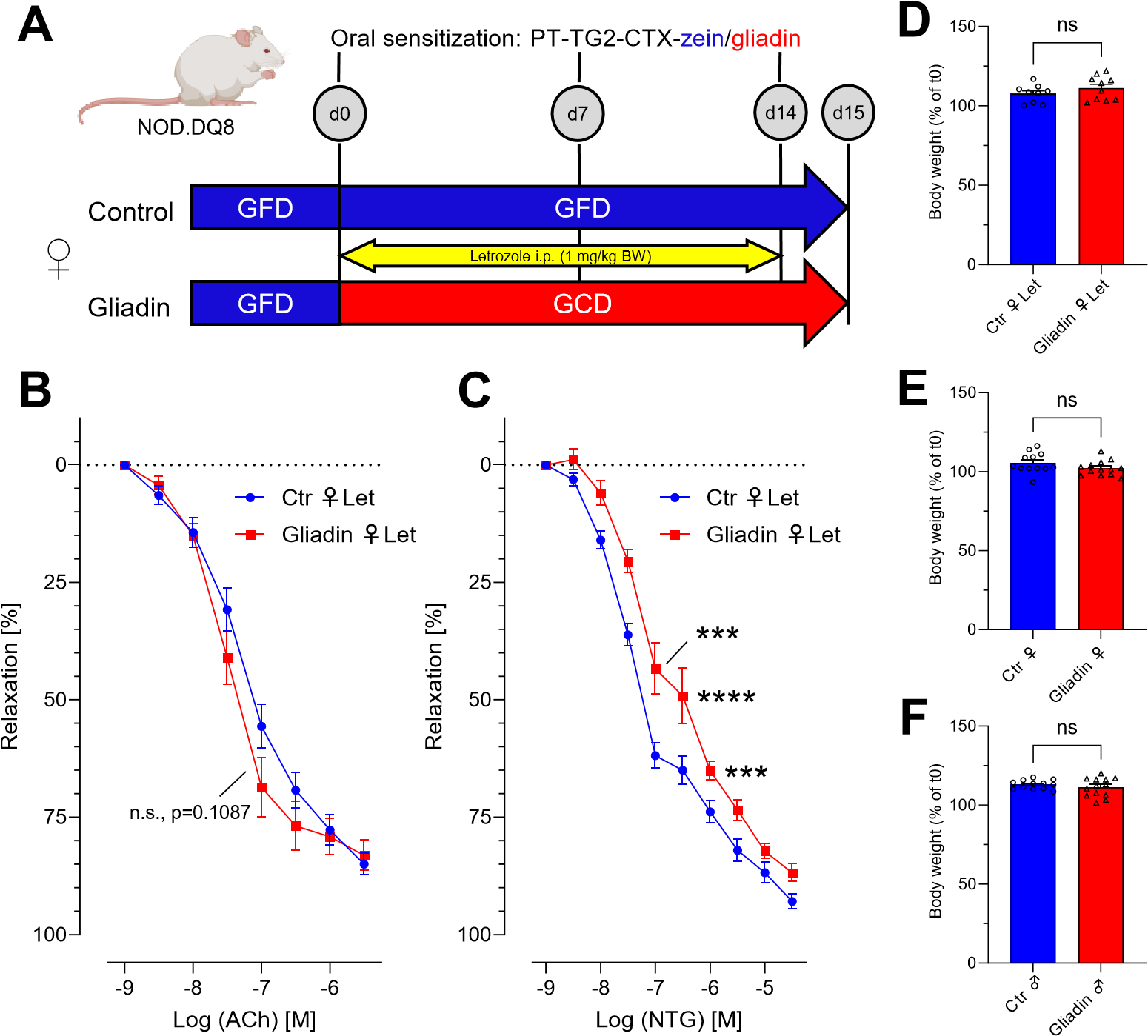


**Supplementary Figure 1: Inhibition of estrogen synthesis in female NOD.DQ8 mice causes endothelium-independent vascular dysfunction.** (A) Females feeding on either GFD or GCD for two weeks were treated with the aromatase inhibitor letrozole by daily intraperitoneal (i.p.) injections (1mg/kg BW) to inhibit estrogen synthesis. (B/C) While endothelium-dependent relaxation capability was not altered between both groups by the letrozole treatment, females exposed to gluten developed an endothelium-independent vascular dysfunction tested by isometric tension studies applying either acetyl-choline (ACh) or nitroglycerine (NTG). (D/E/F) Bodyweight gain over the experimental phase was not altered by the change in diet in any tested group. (B/C) Data shown as arithmetic mean and SD. Two-way ANOVA, n=8; (D-F) Bar graphs show the arithmetic mean and SD. Unpaired t-test, n= 8-9; *** is p<0.001; **** is p<0.0001.

## **A** ✱✱✱✱ **B**


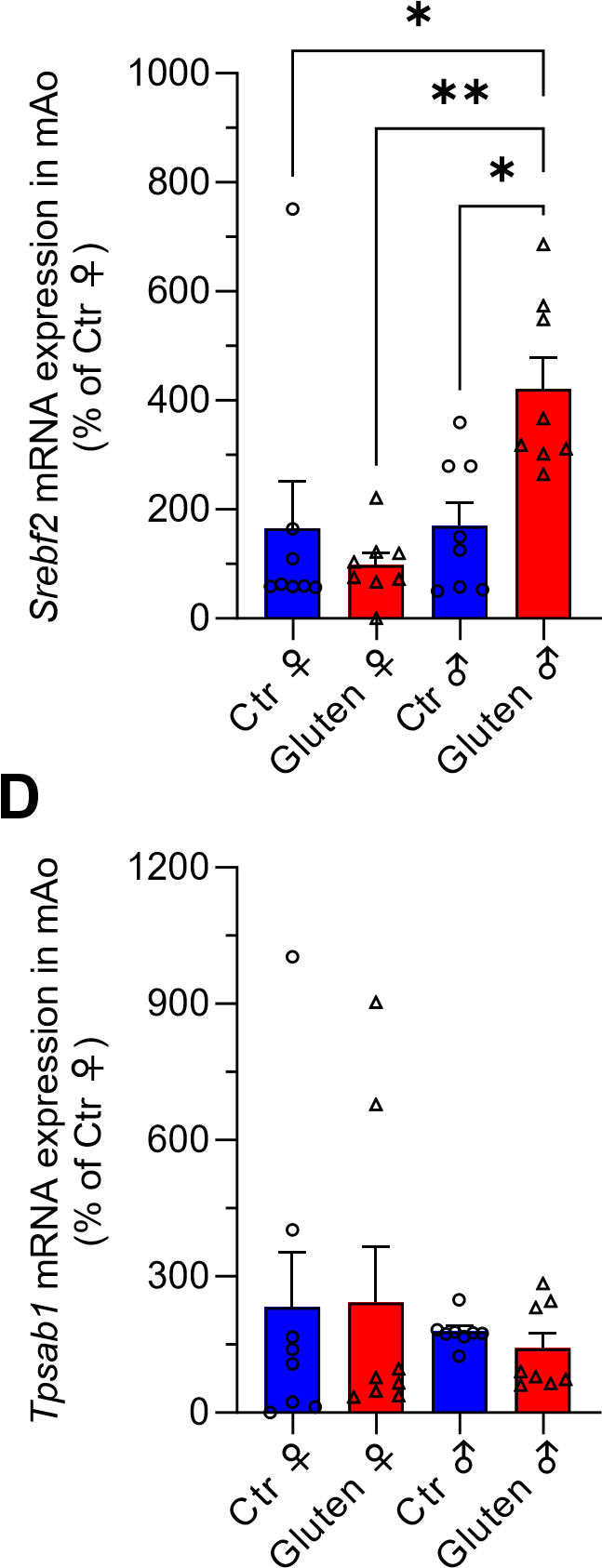

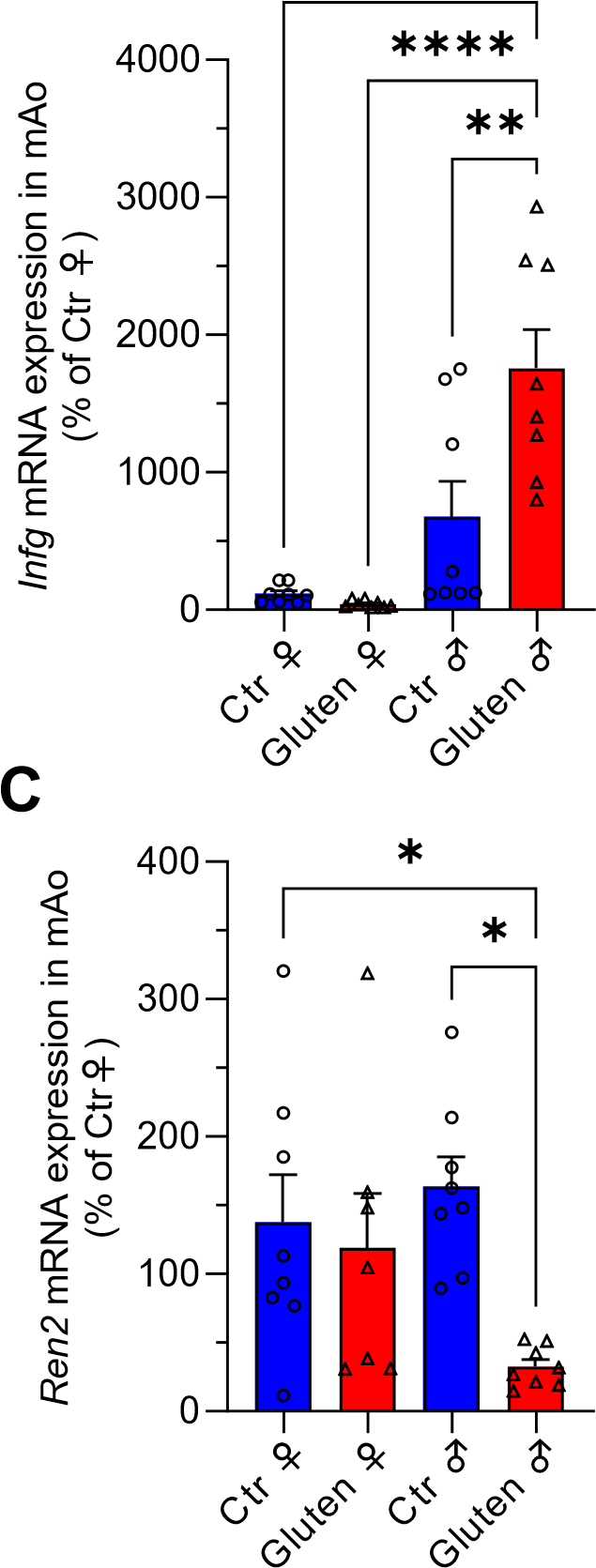


**Supplementary Figure 2: Production of transcripts in aortic specimen which were shown to be altered in pVAT samples.** (A/B) Aortic samples of gluten-exposed males showed increased levels of interferon gamma (Infg) and sterol regulatory element-binding protein 2 (Srebf2). (C/D) Renin 2 (Ren2) expression appeared decreased in gluten males while no alteration was observed for the mast cell marker tryptase alpha/beta-1 (Tpsab1). (A-D) Bar graphs show the arithmetic mean and SD. One-way ANOVA, n=7-8; * is p<0,05, ** is p<0,01, **** is p<0,0001.


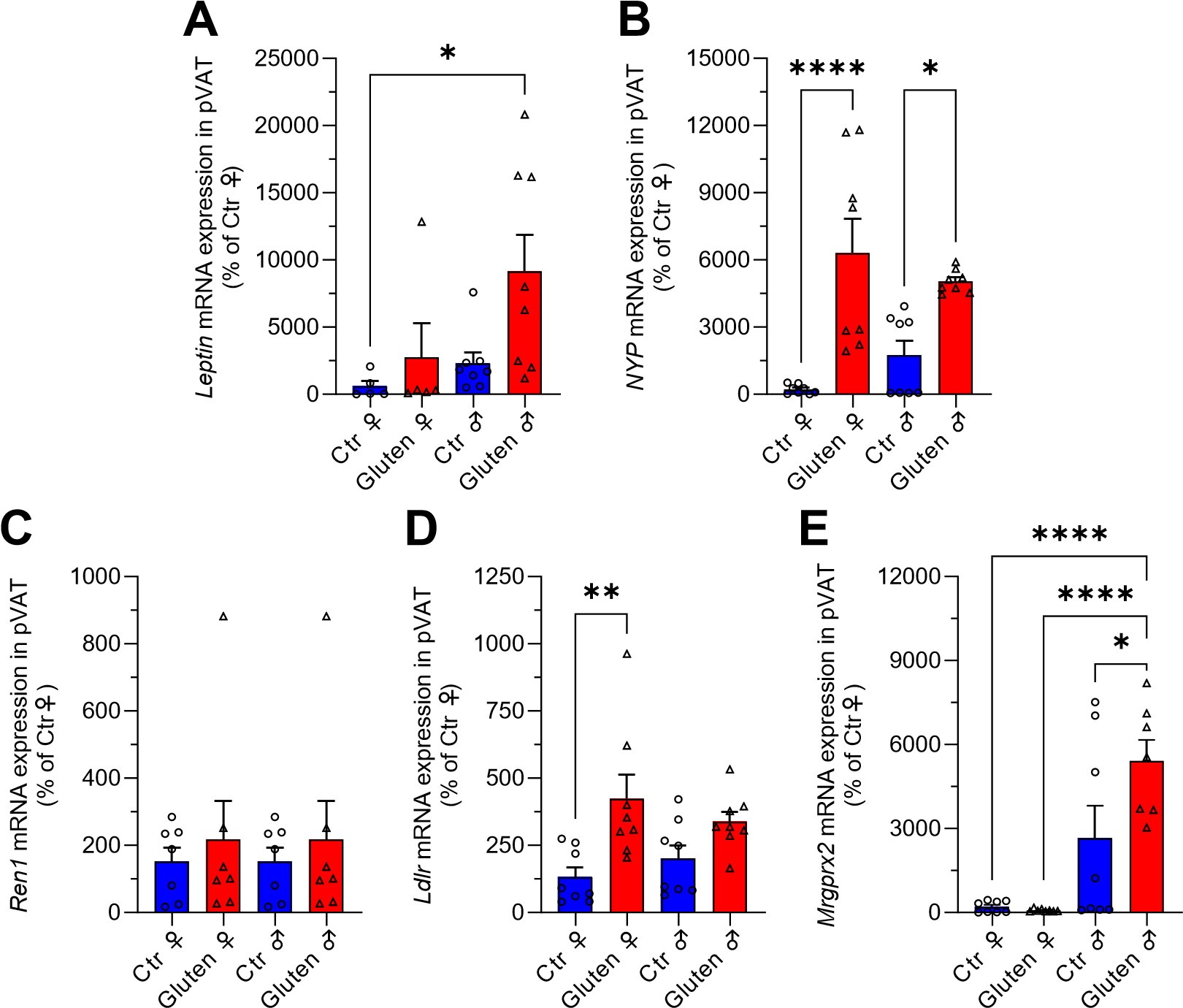


**Supplementary Figure 3: Production of further transcripts in pVAT samples**. (A) Leptin shows a trend towards upregulation in male gliadin-treated mice only. Expression for neuropeptide Y (NPY) was found elevated in both sexes when fed a gluten diet. (C/D) Renin-1 (Ren1) does show a trend to an enhanced expression in both sexes under gluten exposition while LDL-receptor (Ldlr) transcripts were found significantly increased in females only. (E) For Mas-related G-protein coupled receptor member X2 (Mrgprx2), elevated mRNA levels were detected in male mice under gluten exposition. (A-E) Bar graphs show the arithmetic mean and SD. One-way ANOVA, n=78; * is p<0,05, ** is p<0,01, **** is p<0,0001.

**Supplementary Figure 4: Effect sizes by sex and most-altered metabolites.**

(A) To highlight sex-related differences in metabolite expression, the main significant metabolites in the comparison between gluten-fed and control mice were visualized in conjunction with their respective gender counterparts. The y-axis represents the log fold change in females across gluten challenge, while the x-axis displays male gluteninduced changes. The grey diagonal zone indicates metabolites exhibiting comparable fold changes across genders upon gluten challenge, while the white zone delineates differentially expressed metabolites. (B) Metabolites correlating with their expression with 25-hydroxycholesterol in males are presented, indicating whether their appearance matches or correlates negatively. (C) Metabolites correlating with their expression with serotonin in females are depicted, demonstrating whether their appearance matches or correlates negatively.


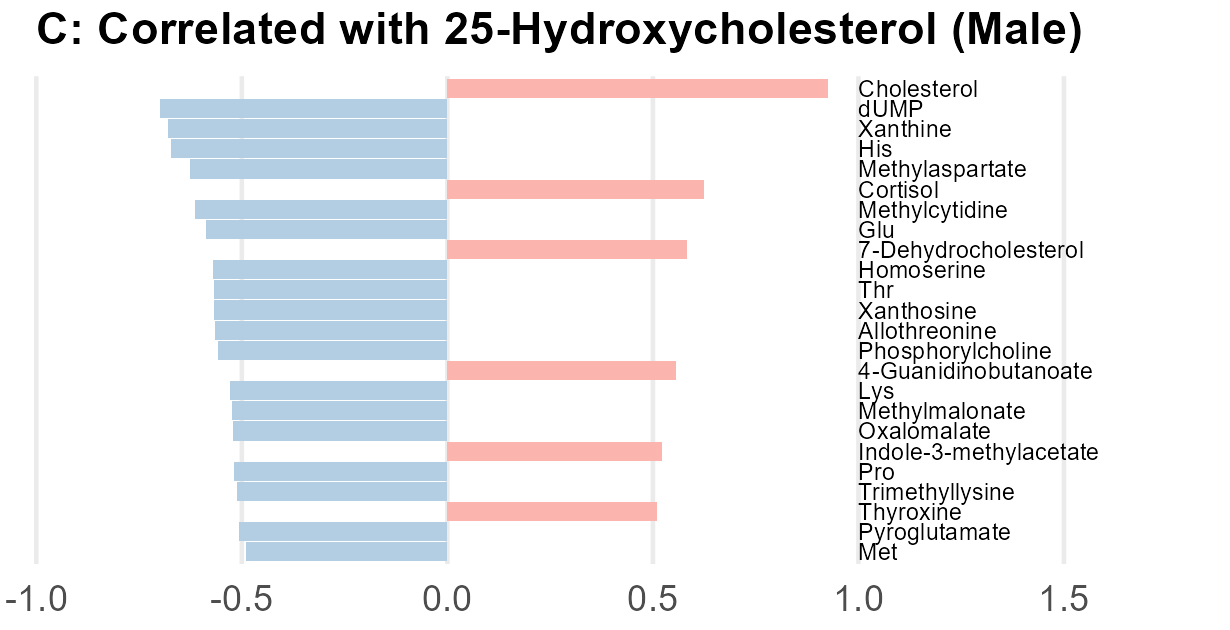

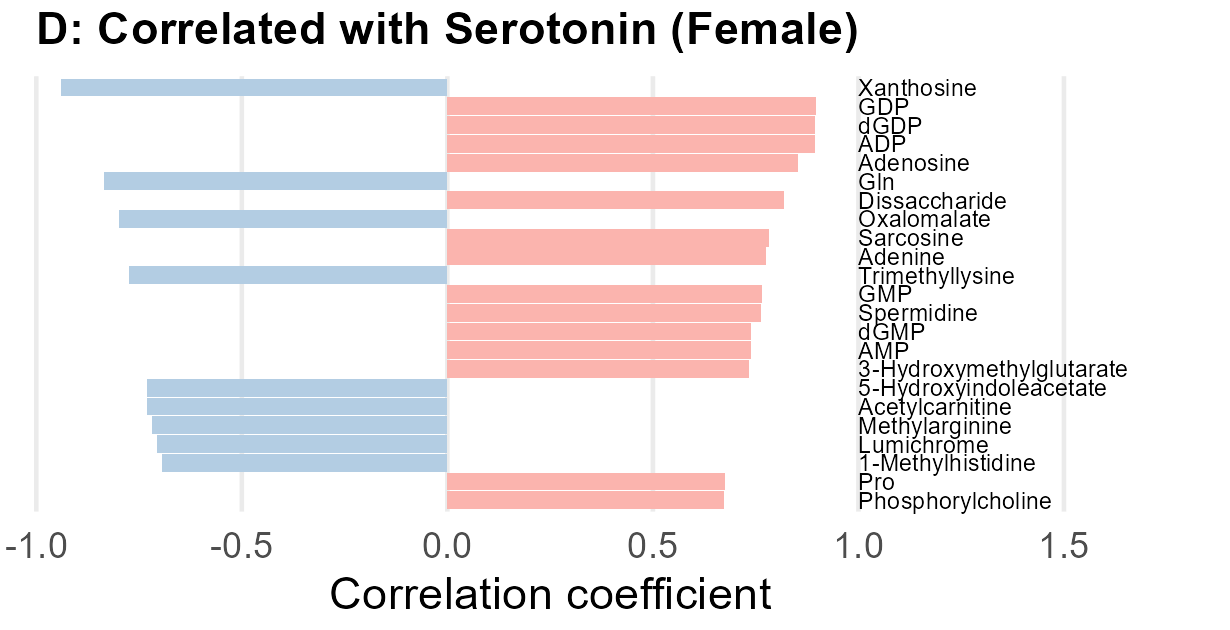

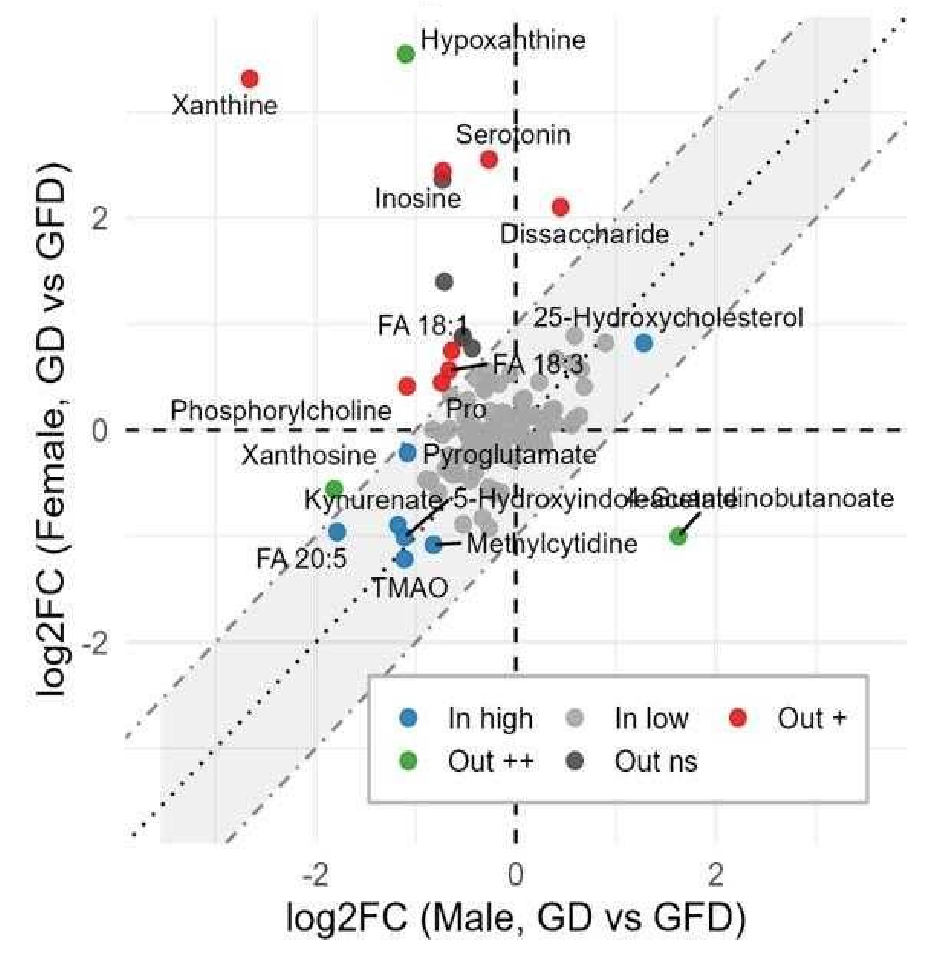


**A**

Effect sizes by sex

**B**

**C**


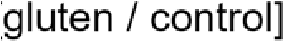

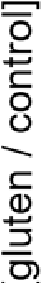

Supplement: Supplementary file 1 — Supplementary Information. [file 41598_2026_45481_MOESM1_ESM.docx]
